# Supplementary material for: Discovery of putative tumor suppressors from CRISPR screens reveals rewired lipid metabolism in acute myeloid leukemia cells
Source: Nat Commun. 2021 Nov 11;12:6506. doi: 10.1038/s41467-021-26867-8 (PMC8586352; doi:10.1038/s41467-021-26867-8)
Supplement: Supplementary file 1 — Supplementary Information [file 41467_2021_26867_MOESM1_ESM.pdf]

Inventory of Supporting Information:

Supplementary Data S1 – Supp_Data_1.zip

Supplementary Data S2 – Supp_Data_2.txt

Supplementary Data S3 – Supp_Data_3.csv

Supplementary Data S4 – Supp_Data_4.xlsx

Supplementary Data S5 – Supp_Data_5.xlsx

Supplementary Data S6 – Supp_Data_6.csv

Supplementary Data S7– Supp_Data_7.csv

Source Data – Source_Data.xlsx

Supplementary Figure 1 – Supp_Fig1.pdf

Supplementary Figure 2 – Supp_Fig2.pdf

Supplementary Figure 3 – Supp_Fig3.pdf

Supplementary Figure 4 – Supp_Fig4.pdf

Supplementary Figure 5 – Supp_Fig5.pdf

Supplementary Figure 6 – Supp_Fig6.pdf

Supplementary Figure 7 – Supp_Fig7.pdf

Supplementary Figure 8 – Supp_Fig8.pdf

Supplementary Figure 9 – Supp_Fig9.pdf

Supplementary Figure 10 – Supp_Fig10.pdf

Supplementary Data

Data S1. Mixed Distribution Model Z-Score Matrix. 808 cell line vs 18,111 gene matrix of mixed Z-score derived from log fold-change fitness scores.

Data S2. COSMIC TSG PS Statistics. Statistics of 116 COSMIC TSG genes when observed as a PS, vs other available data points. Includes number of times TSG is observed as a PS gene (count), mean and median TPM expression when observed as a PS gene and additional backgrounds (PS_Mean_Exp, Other_Mean_Exp, PS_Median_Exp, Other_Median_Exp), and non-silent mutation rate as a PS gene and additional backgrounds (PS_mut, Other_mut). Additionally includes a column of fisher's exact test comparing mutated vs non mutated observations, and a Wilcox test comparing expression levels for each gene.

Data S3. PSG Co-PS network. Network of PSG co-occurrence observations related to **Figures 2c** and **S4c**, including fisher test metrics (p-value and FDR).

Data S4. enCas12a Screen Gene Selection and Rationale. Genes used for genetic interaction screening, and associated rationale of genes.

Data S5. enCas12a Library Design. Guide library used during genetic interaction screening.

Data S6. enCas12a Single Gene Knock-Out Measurements. Z-score of mean Log fold-change.

Data S7. enCas12a Double Gene Knock-Out Measurements. Calculated Log fold-change and corresponding GI Scores for each gene pair.

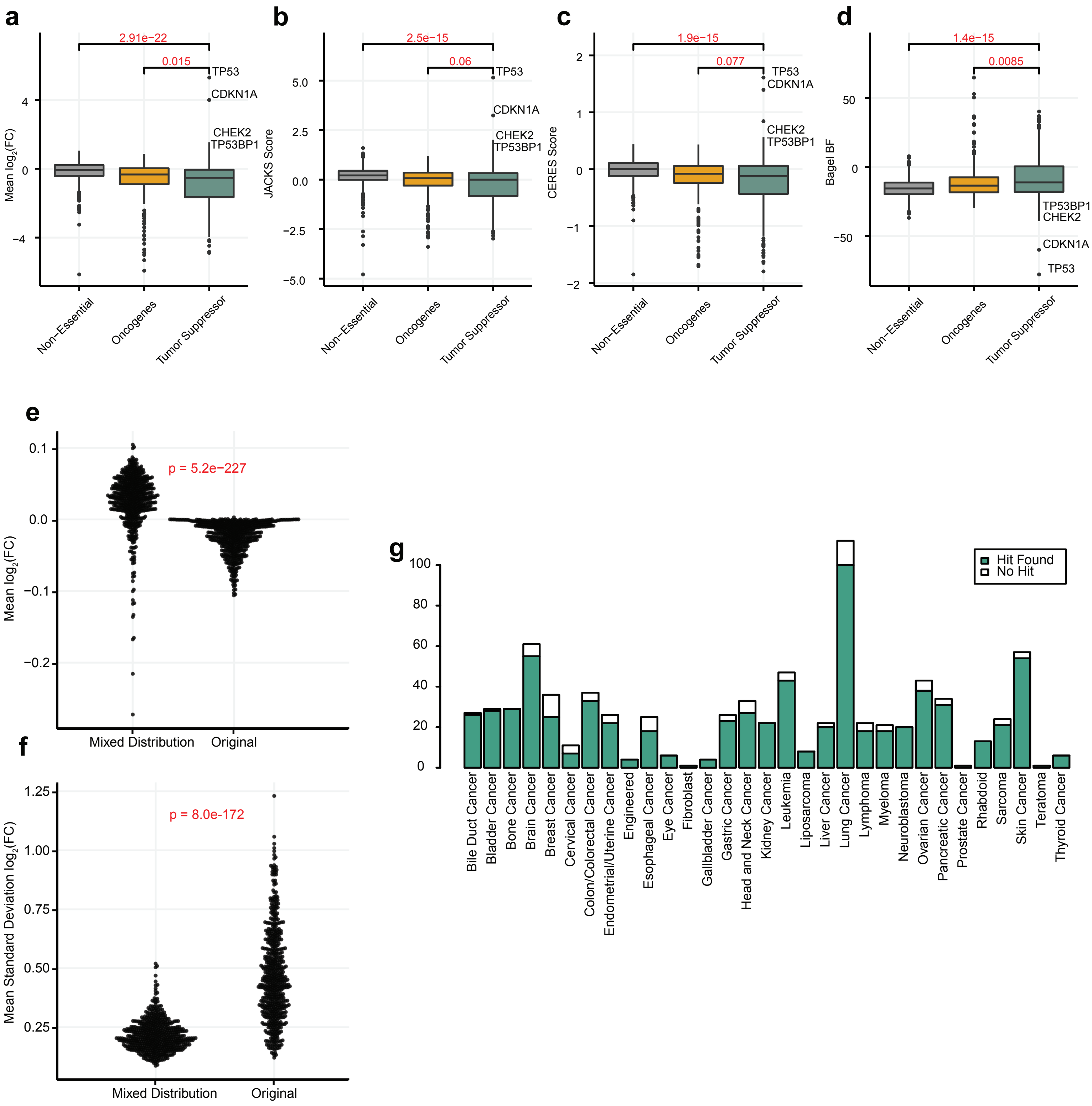
a
2.91e−22
0.015
TP53
CDKN1A
CHEK2
TP53BP1
Mean log2(FC)
4
0
−4
Non−Essential
Oncogenes
Tumor Suppressor
b
2.5e−15
0.06
TP53
CDKN1A
CHEK2
TP53BP1
JACKS Score
5.0
2.5
0.0
−2.5
−5.0
Non−Essential
Oncogenes
Tumor Suppressor
c
1.9e−15
0.077
TP53
CDKN1A
CHEK2
TP53BP1
CERES Score
2
1
0
−1
−2
Non−Essential
Oncogenes
Tumor Suppressor
d
1.4e−15
0.0085
Bagel BF
50
0
−50
TP53BP1
CHEK2
CDKN1A
TP53
Non−Essential
Oncogenes
Tumor Suppressor
e
p = 5.2e−227
Mean log2(FC)
0.1
0.0
−0.1
−0.2
Mixed Distribution
Original
f
p = 8.0e-172
Mean Standard Deviation log2(FC)
1.25
1.00
0.75
0.50
0.25
Mixed Distribution
Original
g
100
80
60
40
20
0
Hit Found
No Hit
Bile Duct Cancer
Bladder Cancer
Bone Cancer
Brain Cancer
Breast Cancer
Cervical Cancer
Colon/Colorectal Cancer
Endometrial/Uterine Cancer
Engineered
Esophageal Cancer
Eye Cancer
Fibroblast
Gallbladder Cancer
Gastric Cancer
Head and Neck Cancer
Kidney Cancer
Leukemia
Liposarcoma
Liver Cancer
Lung Cancer
Lymphoma
Myeloma
Neuroblastoma
Ovarian Cancer
Pancreatic Cancer
Prostate Cancer
Rhabdoid
Sarcoma
Skin Cancer
Teratoma
Thyroid Cancer

Supplementary figure legends

Figure S1. Discovery of Proliferation Suppressor genes extended. Fitness scoring distributions of non-essential genes, and non-overlapping COSMIC defined oncogenes and tumor suppressor genes; (a) mean log fold-change, Non-Essential min = -6.14, max = 1.06, Q1-1.5*IQR (lower whisker) = -1.32, 25th percentile (%) = -0.41, median = -0.07, 75th% = 0.21, Q3+1.5IQR (upper whisker) = 1.06, Oncogenes min = -5.92, max = 0.87, lower whisker = -2.04, 25th%= -0.90, median = -0.33, 75th% = 0.04, upper whisker = 0.87, Tumor Suppressors min = -4.89, max = 5.30, lower whisker = -3.96, 25th% = -1.65, median = -0.52, 75th%= -0.05, upper whisker = 1.55, (b) JACKS, Non-Essential min = -4.80 , max = 1.60, lower whisker = -0.69, 25th% = -0.01, median = 0.21, 75th%= 0.45, upper whisker = 1.13, Oncogenes min = -3.39, max = 1.19, lower whisker = -1.28, 25th% = -0.30, median = 0.07, 75th% = 0.35, upper whisker = 1.19, Tumor Suppressors min = -2.98, max = 5.15, lower whisker = -2.55, 25th% = -0.83, median = 0.00, 75th% = 0.33, upper whisker = 2.02, (c) CERES, Non-Essential min = -1.85 , max = 0.43, lower whisker = -0.44, 25th% = -0.12, median = 0.00, 75th% = 0.11, upper whisker = 0.43, Oncogenes min = -1.71, max = 0.43, lower whisker = -0.62, 25th% = -0.24, median = -0.08, 75th% = 0.05upper whisker = 0.43, Tumor Suppressors min = -1.79, max = 1.62, lower whisker = -1.17, 25th% = -0.43, median = -0.12, 75th% = 0.06, upper whisker = 0.56, and (d) BAGEL, Non-Essential min = -36.83 , max = 7.82, lower whisker = -31.91, 25th% = -19.66, median = -15.54, 75th% = -11.27, upper whisker = 1.25, Oncogenes min = -29.64, max = 64.871, lower whisker = -29.64, 25th% = -18.35, median = -13.48, 75th% = -7.50, upper whisker = 8.22, Tumor Suppressors min = -77.92, max = 40.26, lower whisker = -39.20, 25th% = -17.95, median = -11.15, 75th% = 0.48, upper whisker = 27.80. Boxplots contain identical number of genes with “Non-Essential” genes (n = 819 unique genes), Oncogenes (n =

236 unique genes), and Tumor Suppressors (n = 311 unique genes). P values indicate significance testing of two-sided Wilcoxon rank-sum test. Selected screen for a-d matches the single screen observed in Figure 1a. (e) Distribution of mean log fold-change of original distribution and mixed distribution. P values indicate significance testing of a two-sided Wilcoxon rank-sum test. (f) Same (e) with mean standard deviation. (g) Bar chart by cell line lineage, where at least 1 PS gene at 10% FDR cutoff identified.

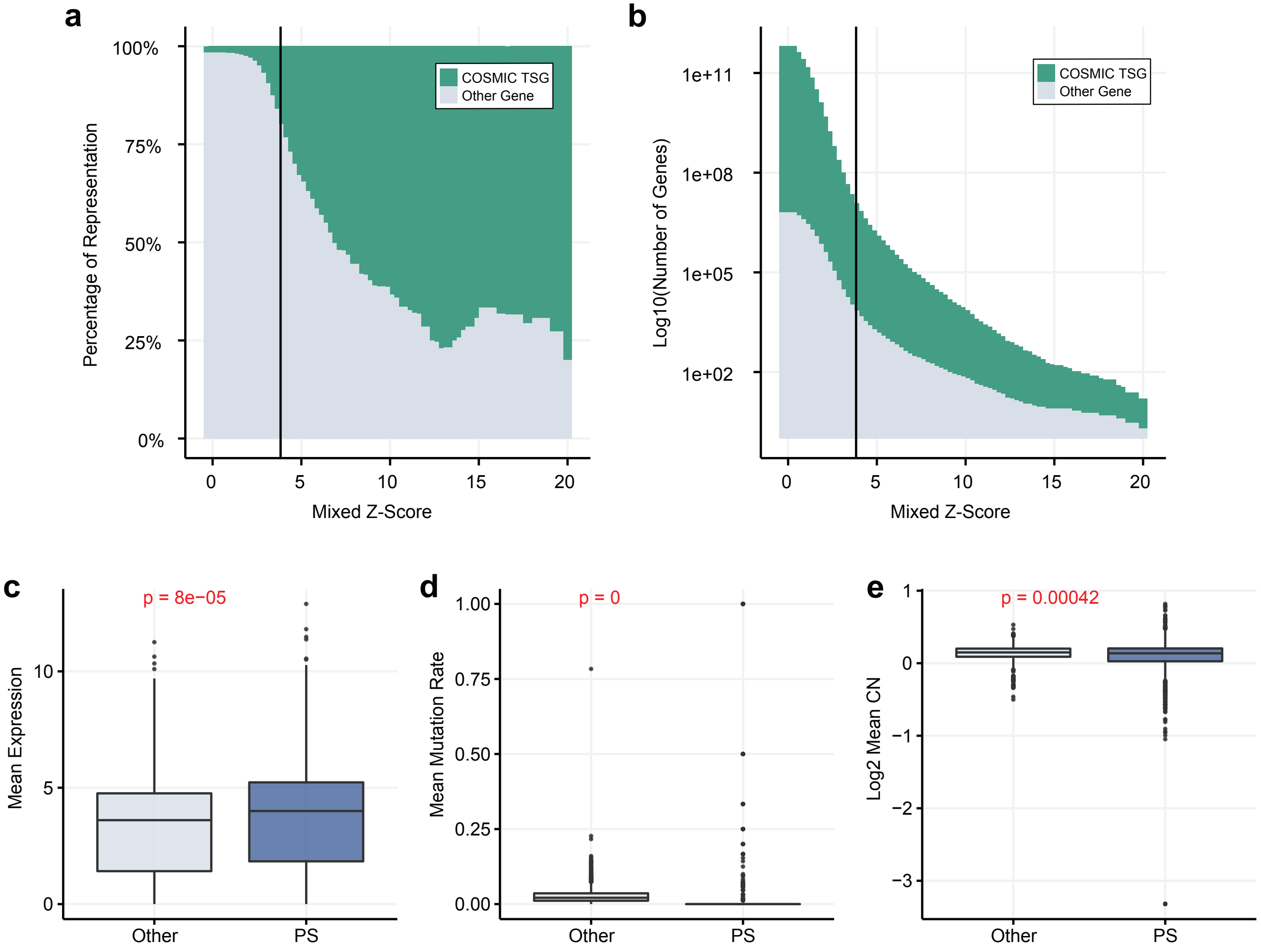
a
100%
75%
50%
25%
0%
Percentage of Representation
COSMIC TSG
Other Gene
0
5
10
15
20
Mixed Z-Score
b
1e+11
1e+08
1e+05
1e+02
Log10(Number of Genes)
COSMIC TSG
Other Gene
0
5
10
15
20
Mixed Z-Score
c
p = 8e−05
Mean Expression
10
5
0
Other
PS
d
p = 0
Mean Mutation Rate
1.00
0.75
0.50
0.25
0.00
Other
PS
e
p = 0.00042
Log2 Mean CN
1
0
−1
−2
−3
Other
PS

Figure S2. Proliferation Suppressor Gene Evidence. (a) Percent representation of COSMIC TSG (green) by corresponding mixed Z-score. (b) Same as (a) with log10 y-axis of number of genes. (c) Mean TPM expression of PSG, grouped by PS observations (blue) vs every other available observation (gray) in which PSG were not observed as a PS. P value represents the corresponding two-sided Wilcoxon rank-sum test. PS observations (blue), n = 1,280 unique genes, min = 0, max = 12.90, Q1-1.5*IQR (lower whisker) = 0, 25th percentile (%) = 1.84, median = 4.00, 75th% = 5.23, Q3+1.5IQR (upper whisker) = 10.27. 'Other' observations (gray), n = 1453 unique genes, min = 0, max = 11.25, lower whisker = 0, 25th% = 1.42, median = 3.61, 75th% = 4.76, upper whisker = 9.69. (d) same as (c) with mutation rate PS observations (blue), n = 1,483 unique genes, min = 0, max = 1, boxplot statistics all = 0, 'Other' observations (gray), n = 1483 unique genes, min = 0, max = 0.78, lower whisker = 0, 25th% = 0.01, median = 0.02, 75th% = 0.04, upper whisker = 0.07 and (e) copy number PS observations (blue), n = 1,435 unique genes, min = 1.41e-09, max = 1.66, lower whisker = 0.72, 25th% = 0.92, median = 1.00, 75th% = 1.05, upper whisker = 1.25, 'Other' observations (gray), n = 1457 unique genes, min = 0.61, max = 1.34, lower whisker = 0.83, 25th% = 0.96, median = 1.01, 75th% = 1.05, upper whisker = 1.18.

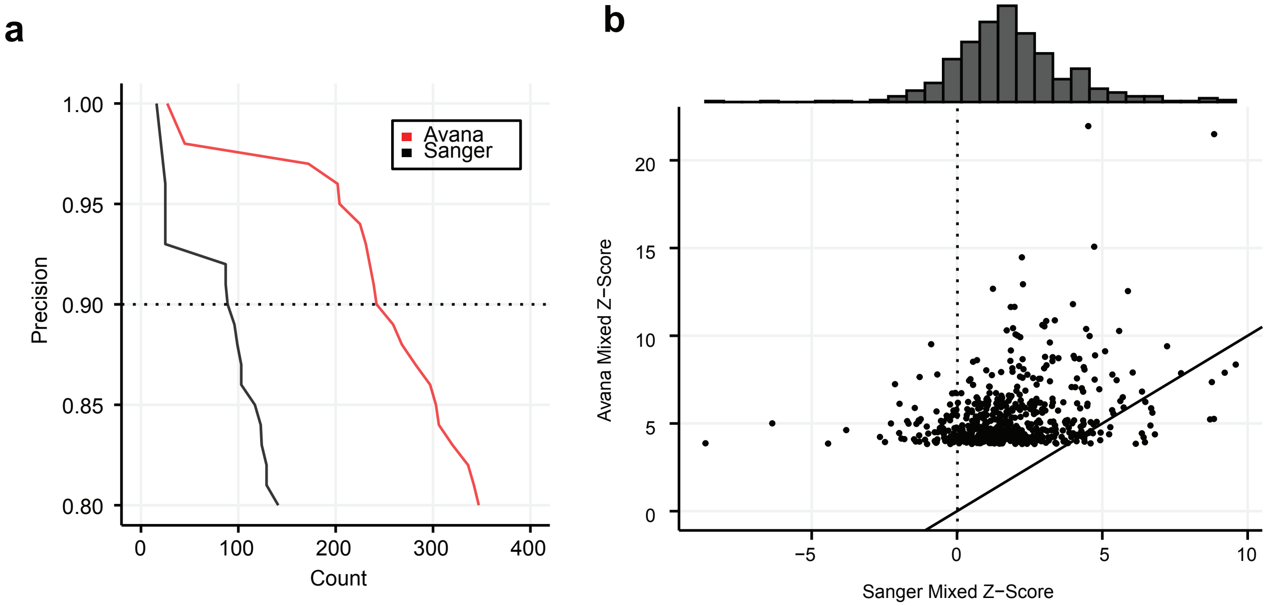
a
b
Avana
Sanger
Precision
1.00
0.95
0.90
0.85
0.80
0
100
200
300
400
Count
Avana Mixed Z−Score
Sanger Mixed Z−Score
20
15
10
5
0
−5
0
5
10

Figure S3. Avana vs Sanger Genetic Screens Comparison. (a) Precision vs. count recall of mixed Z-score in matching screens from Avana (red), and Sanger (black). Dashed line represents 90% precision (10% FDR). (b) Avana vs Sanger mixed Z-scores of genes identified as hits in Avana. Dashed line indicates Sanger mixed z-score = 0.

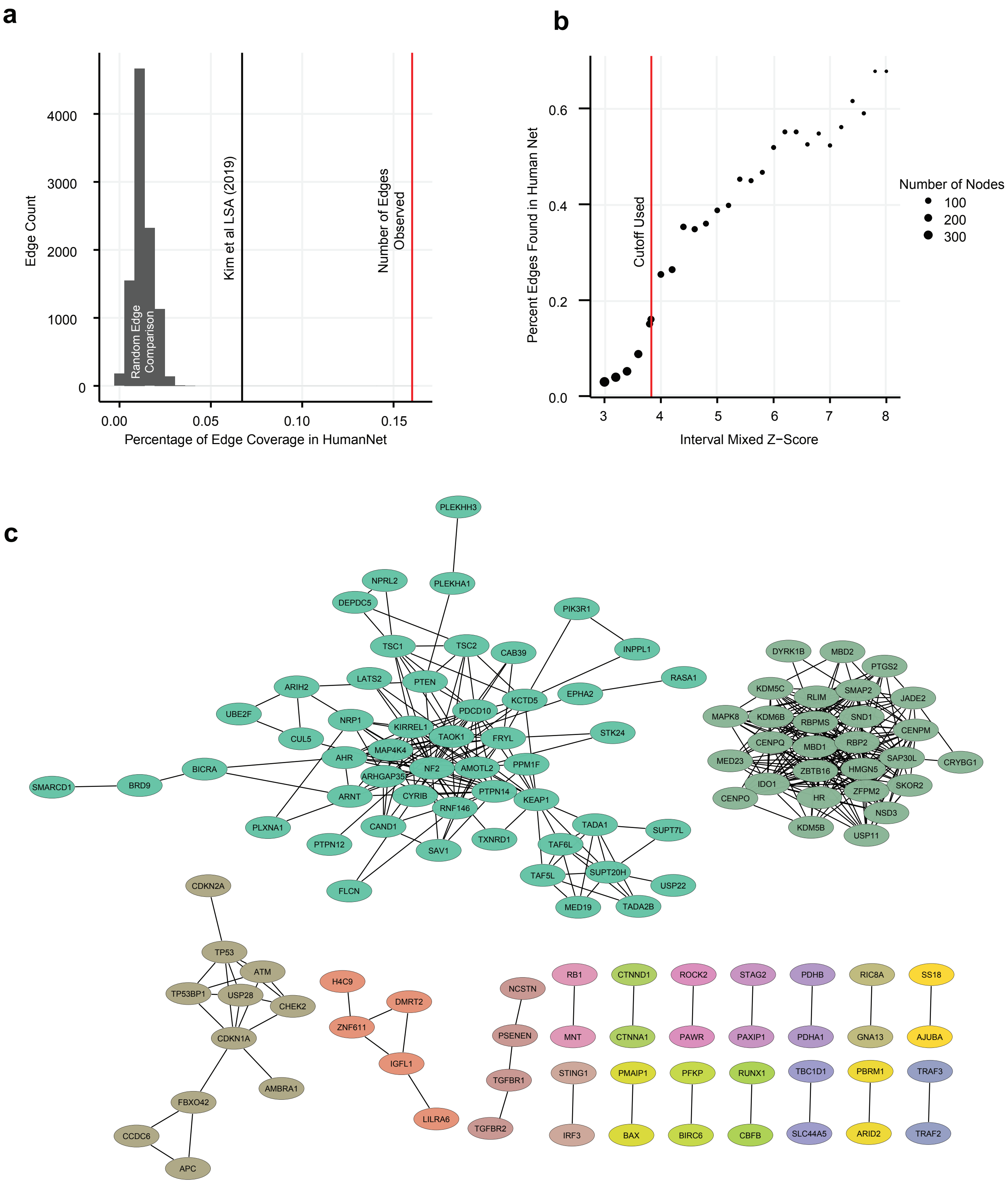
a
Edge Count
4000
3000
2000
1000
0
0.00
0.05
0.10
0.15
Percentage of Edge Coverage in HumanNet
Random Edge Comparison
Kim et al LSA (2019)
Number of Edges Observed
b
Percent Edges Found in Human Net
0.6
0.4
0.2
0.0
3
4
5
6
7
8
Interval Mixed Z−Score
Cutoff Used
Number of Nodes
100
200
300
c
PLEKHH3
NPRL2
PLEKHA1
DEPDC5
PIK3R1
INPPL1
TSC1
TSC2
CAB39
RASA1
LATS2
PTEN
KCTD5
EPHA2
ARIH2
PDCD10
UBE2F
NRP1
KIRREL1
STK24
CUL5
TAOK1
FRYL
AHR
MAP4K4
PPM1F
BICRA
NF2
AMOTL2
ARHGAP35
SMARCD1
BRD9
ARNT
CYRIB
PTPN14
KEAP1
RNF146
PLXNA1
CAND1
TADA1
SUPT7L
PTPN12
SAV1
TXNRD1
TAF6L
SUPT20H
TAF5L
USP22
FLCN
MED19
TADA2B
DYRK1B
MBD2
PTGS2
KDM5C
RLIM
SMAP2
JADE2
MAPK8
KDM6B
RBPMS
SND1
CENPM
CENPQ
MBD1
RBP2
MED23
SAP30L
CRYBG1
ZBTB16
HMGN5
IDO1
SKOR2
CENPO
HR
ZFPM2
NSD3
KDM5B
USP11
CDKN2A
TP53
ATM
TP53BP1
USP28
CHEK2
CDKN1A
AMBRA1
FBXO42
CCDC6
APC
H4C9
DMRT2
ZNF611
IGFL1
LILRA6
NCSTN
PSENEN
TGFBR1
TGFBR2
RB1
MNT
STING1
IRF3
CTNND1
CTNNA1
PMAIP1
BAX
ROCK2
PAWR
PFKP
BIRC6
STAG2
PAXIP1
RUNX1
CBFB
PDHB
PDHA1
TBC1D1
SLC44A5
RIC8A
GNA13
PBRM1
ARID2
SS18
AJUBA
TRAF3
TRAF2

Figure S4. Co-occurrence of PS genes extended. (a) Empirical comparison of Co-PS network edges. Distribution represents random edges between genes identified in the network, and the percentage of edges identified in HumanNet with coessentiality network removed. Black line represents the percent of edges identified in the Kim *et al.* coessentiality network. Red line indicates the actual number of edges the Co-PS contains that are observed in HumanNet with coessentiality network removed. (b) Percent of edge coverage observed in HumanNet with coessentiality network removed against Co-PS edge FDR < 0.1% networks at iterative label mixed Z-score cutoffs. Red line indicates actual cutoff used. (c) Remaining modules from the Co-PS network not included in Figure 2c. Clusters are colored uniquely to demonstrate distinctions between gene modules.

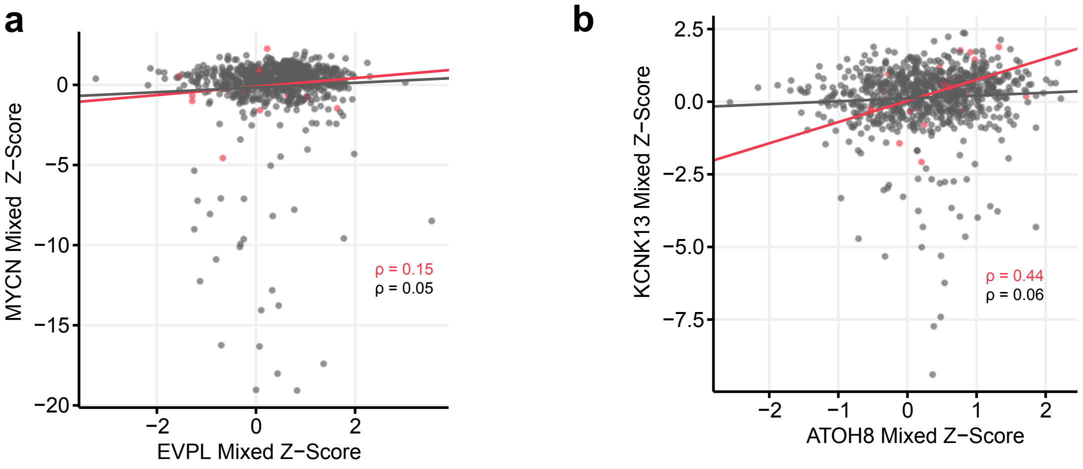
a
MYCN Mixed Z−Score
EVPL Mixed Z−Score
0
−5
−10
−15
−20
−2
0
2
ρ = 0.15
ρ = 0.05
b
KCNK13 Mixed Z−Score
ATOH8 Mixed Z−Score
2.5
0.0
−2.5
−5.0
−7.5
−2
−1
0
1
2
ρ = 0.44
ρ = 0.06

Figure S5. Examples of high dPCC resulting from data noise. (a) EVPL vs MYCN mixed Z-scores. Red indicates AML only observations, while gray indicates observations in all other cells. (b) same as (a) for ATOH8 vs. KNCK13 mixed Z-scores. Values plotted represent correlation (ρ) of plotted points.

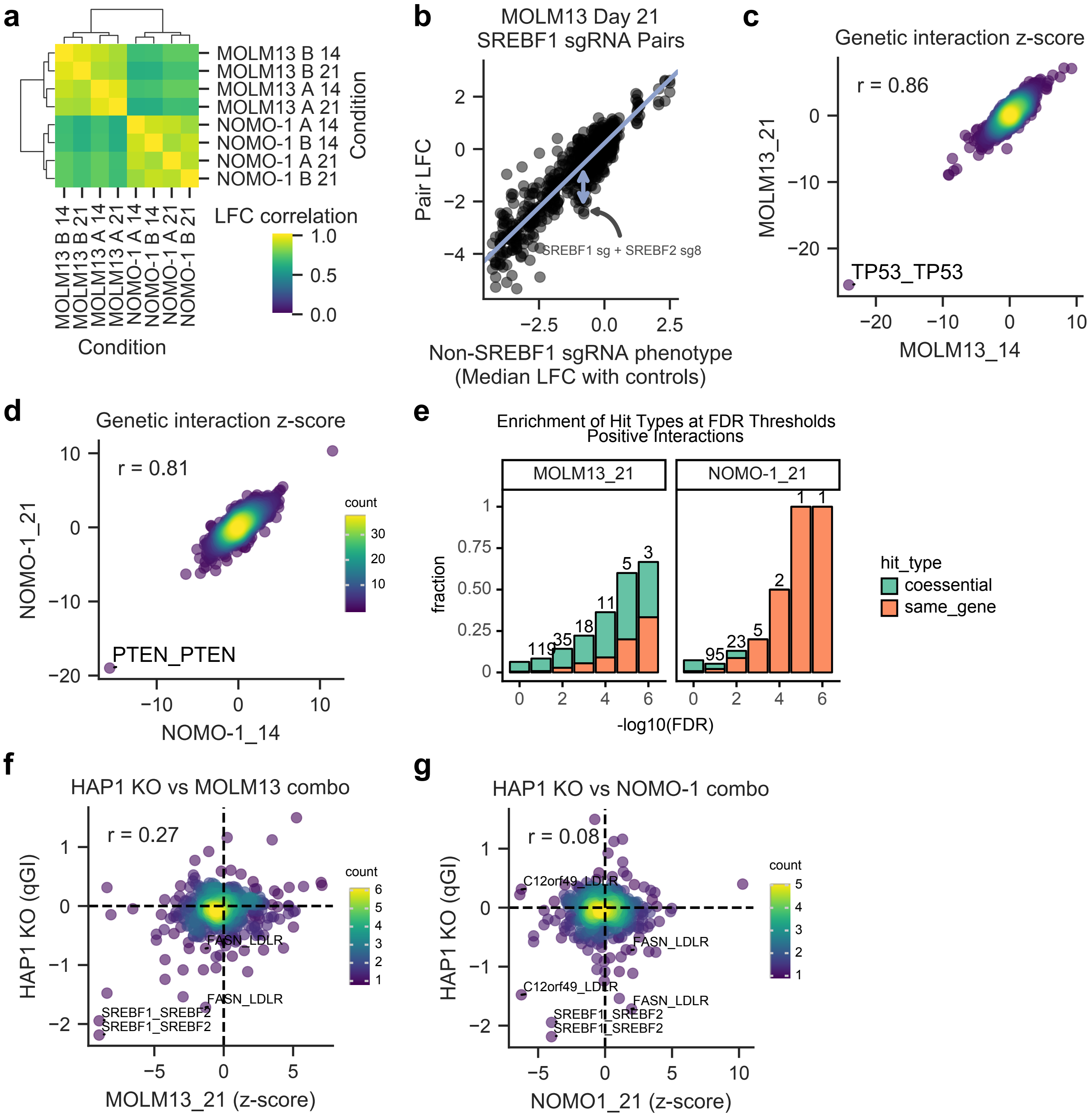
a
MOLM13 B 14
MOLM13 B 21
MOLM13 A 14
MOLM13 A 21
NOMO-1 A 14
NOMO-1 B 14
NOMO-1 A 21
NOMO-1 B 21
Condition
LFC correlation
1.0
0.5
0.0
b
MOLM13 Day 21
SREBF1 sgRNA Pairs
Pair LFC
SREBF1 sg + SREBF2 sg8
Non-SREBF1 sgRNA phenotype
(Median LFC with controls)
c
Genetic interaction z-score
r = 0.86
MOLM13_21
MOLM13_14
TP53_TP53
count
d
Genetic interaction z-score
r = 0.81
NOMO-1_21
NOMO-1_14
PTEN_PTEN
count
e
Enrichment of Hit Types at FDR Thresholds
Positive Interactions
MOLM13_21
NOMO-1_21
fraction
-log10(FDR)
hit_type
coessential
same_gene
f
HAP1 KO vs MOLM13 combo
r = 0.27
HAP1 KO (qGI)
MOLM13_21 (z-score)
FASN_LDLR
SREBF1_SREBF2
count
g
HAP1 KO vs NOMO-1 combo
r = 0.08
HAP1 KO (qGI)
NOMO1_21 (z-score)
C12orf49_LDLR
FASN_LDLR
SREBF1_SREBF2
count

Figure S6. Combinatorial screen QC. (a) Replicate correlations. (b) Example calculation of residuals. (c) Correlation between genetic interaction scores for MOLM13. (d) same as (c) for NOMO1. (e) Fraction of coessential pairs or pairs that target the same gene at different FDR cutoffs for interactions with positive z-scores. (f) Comparison with qGI scores from Aregger *et al.*[1] for MOLM13. (g) Same as (f) for NOMO1.

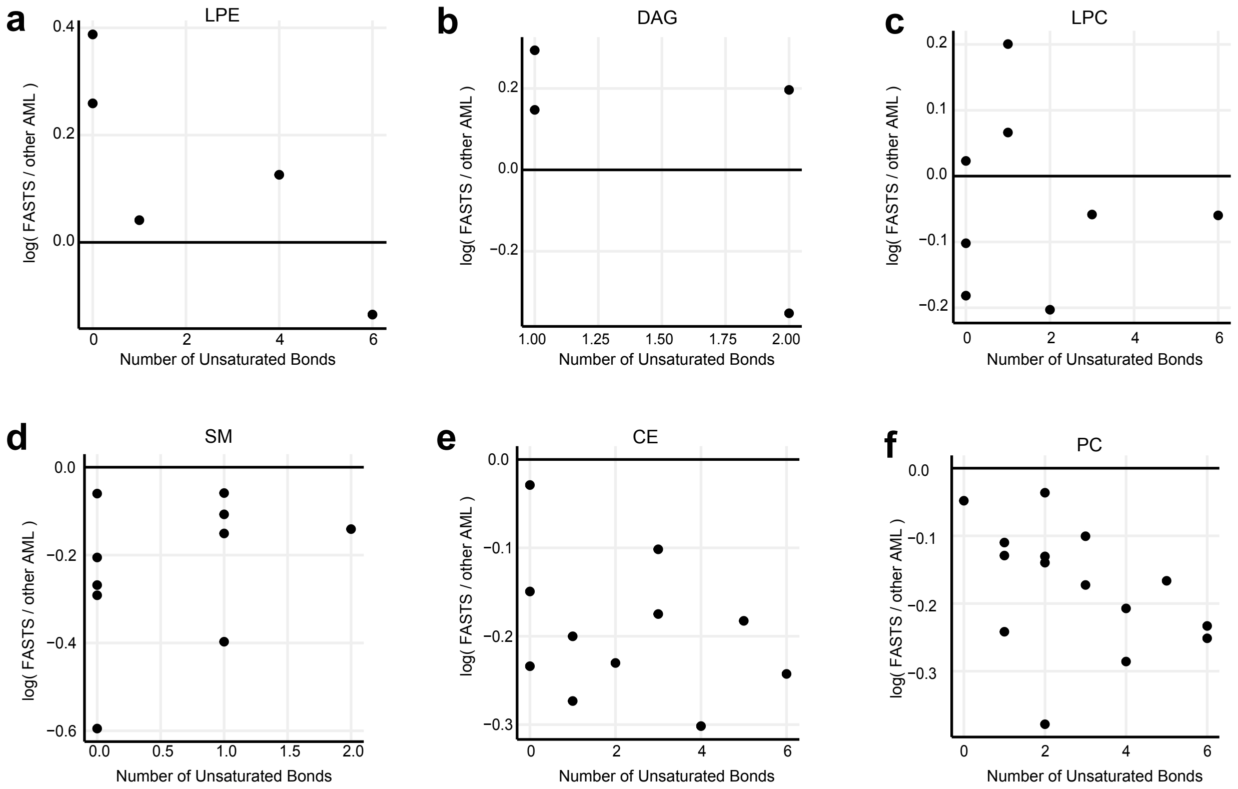

a
LPE
b
DAG
c
LPC
d
SM
e
CE
f
PC
log(FASTS / other AML)
Number of Unsaturated Bonds

Figure S7. Additional metabolite comparisons. (a) Lysophosphatidylethanolamine (LPE) species metabolite difference. The x axis represents the median difference of log10 normalized peak area of the metabolite in FASTS cells vs all other AML cells. The y axis represents the number of saturated bonds present. Each dot represents a unique metabolite. (b) same for diacylglycerol (DAG), (c) lysophosphatidylcholine (LPC), (d) sphingomyelin (SM), (e) cholesterol ester (CE), and (f) phosphatidylcholine (PC) species.

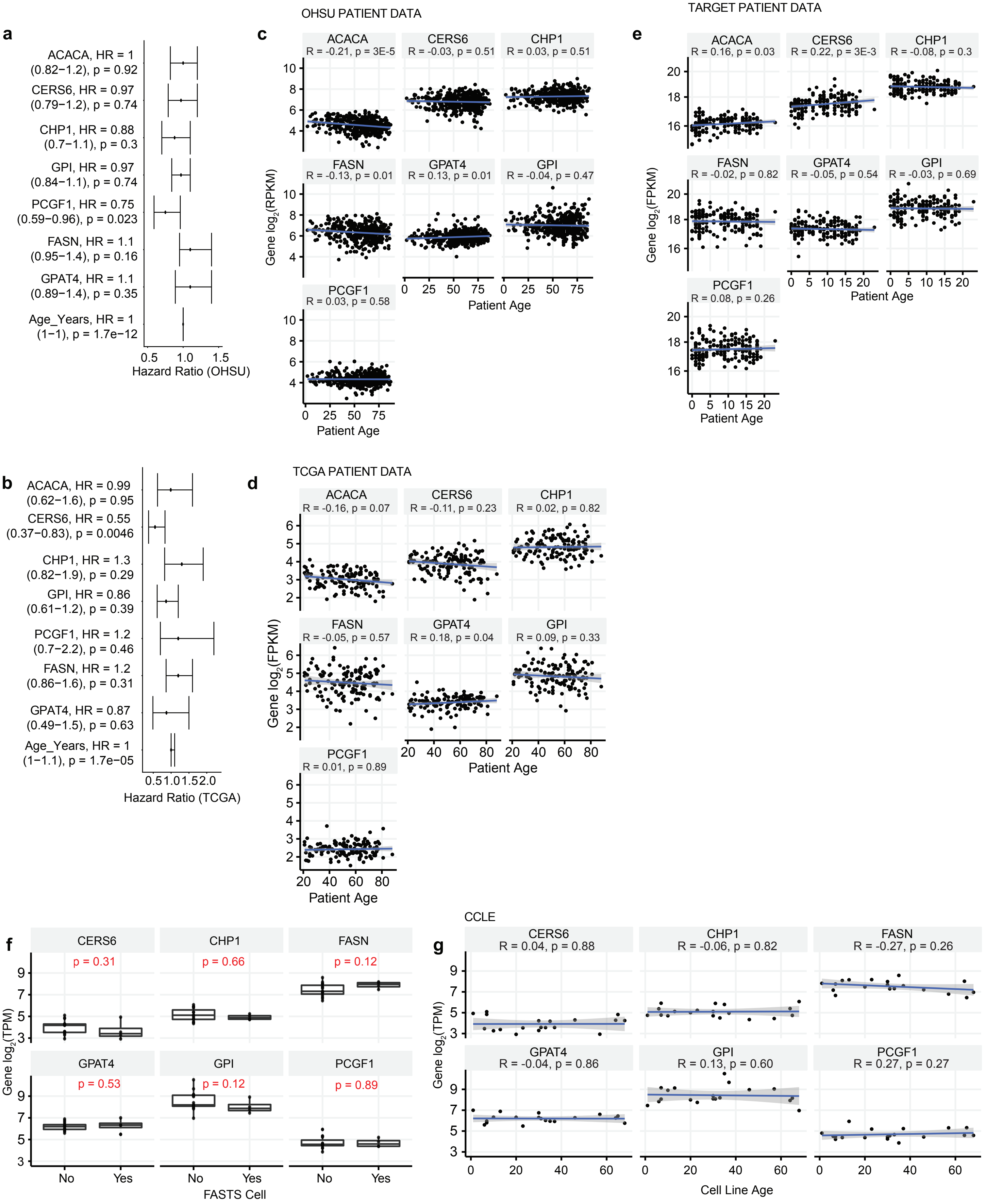
OHSU PATIENT DATA
TARGET PATIENT DATA
a
ACACA, HR = 1 (0.82−1.2), p = 0.92
CERS6, HR = 0.97 (0.79−1.2), p = 0.74
CHP1, HR = 0.88 (0.7−1.1), p = 0.3
GPI, HR = 0.97 (0.84−1.1), p = 0.74
PCGF1, HR = 0.75 (0.59−0.96), p = 0.023
FASN, HR = 1.1 (0.95−1.4), p = 0.16
GPAT4, HR = 1.1 (0.89−1.4), p = 0.35
Age_Years, HR = 1 (1−1), p = 1.7e−12
Hazard Ratio (OHSU)
c
ACACA R = -0.21, p = 3E-5
CERS6 R = -0.03, p = 0.51
CHP1 R = 0.03, p = 0.51
FASN R = -0.13, p = 0.01
GPAT4 R = 0.13, p = 0.01
GPI R = -0.04, p = 0.47
PCGF1 R = 0.03, p = 0.58
Gene log2(RPKM)
Patient Age
e
ACACA R = 0.16, p = 0.03
CERS6 R = 0.22, p = 3E-3
CHP1 R = -0.08, p = 0.3
FASN R = -0.02, p = 0.82
GPAT4 R = -0.05, p = 0.54
GPI R = -0.03, p = 0.69
PCGF1 R = 0.08, p = 0.26
Gene log2(FPKM)
Patient Age
b
ACACA, HR = 0.99 (0.62−1.6), p = 0.95
CERS6, HR = 0.55 (0.37−0.83), p = 0.0046
CHP1, HR = 1.3 (0.82−1.9), p = 0.29
GPI, HR = 0.86 (0.61−1.2), p = 0.39
PCGF1, HR = 1.2 (0.7−2.2), p = 0.46
FASN, HR = 1.2 (0.86−1.6), p = 0.31
GPAT4, HR = 0.87 (0.49−1.5), p = 0.63
Age_Years, HR = 1 (1−1.1), p = 1.7e−05
Hazard Ratio (TCGA)
TCGA PATIENT DATA
d
ACACA R = -0.16, p = 0.07
CERS6 R = -0.11, p = 0.23
CHP1 R = 0.02, p = 0.82
FASN R = -0.05, p = 0.57
GPAT4 R = 0.18, p = 0.04
GPI R = 0.09, p = 0.33
PCGF1 R = 0.01, p = 0.89
Gene log2(FPKM)
Patient Age
f
CERS6 p = 0.31
CHP1 p = 0.66
FASN p = 0.12
GPAT4 p = 0.53
GPI p = 0.12
PCGF1 p = 0.89
Gene log2(TPM)
No Yes
FASTS Cell
CCLE
g
CERS6 R = 0.04, p = 0.88
CHP1 R = -0.06, p = 0.82
FASN R = -0.27, p = 0.26
GPAT4 R = -0.04, p = 0.86
GPI R = 0.13, p = 0.60
PCGF1 R = 0.27, p = 0.27
Gene log2(TPM)
Cell Line Age

Figure S8. Comparisons of FAS genes against age in AML patient data. Hazard ratio calculations for FAS cluster genes in AML patient data coming from (a) OHSU - Tyner *et al.,* and (b) TCGA LAML. The dots plotted in S8a and S8b represents the HR value indicated on the right. The 95% CI range for each point is indicated on the left. Spearman correlations of patient age against FAS gene expression in (c) OHSU, Tyner *et al.,* (d) TCGA LAML, and (e) GDC TARGET AML. (f) Boxplots of FAS gene expression in FASTS AML, n = 4 biologically independent cell lines, and non-FASTS AML, n = 15 biologically independent cell lines from CCLE. All p-values denote two-sided Wilcoxon rank-sum test. For *CERS6* FASTS = No, min = 2.94, max = 5.10, Q1-1.5*IQR (lower whisker) = 2.94, 25th percentile (%) = 3.52, median = 4.18, 75th% = 4.29, Q3+1.5IQR (upper whisker) = 5.10, FASTS = Yes, min = 2.92, max = 4.93, lower whisker = 2.92, 25th% = 3.09, median = 3.41, 75th% = 4.24, upper whisker = 4.93. For *CHP1* FASTS = No, min = 4.34, max = 6.07, lower whisker = 4.34, 25th% = 4.73, median = 5.11, 75th% = 5.60, upper whisker = 6.07, FASTS = Yes, min = 4.71, max = 5.23, lower whisker = 4.71, 25th% = 4.73, median = 4.88, 75th% = 5.12, upper whisker = 5.23. For *FASN* FASTS = No, min = 6.45, max = 8.58, lower whisker = 6.45, 25th% = 7.06, median = 7.31, 75th% = 7.87, upper whisker = 8.58, FASTS = Yes, min = 7.48, max = 8.17, lower whisker = 7.48, 25th% = 7.65, median = 7.95, 75th% = 8.13, upper whisker = 8.17. For *GPAT4* FASTS = No, min = 5.59, max = 6.89, lower whisker = 5.59, 25th% = 5.94, median = 6.19, 75th% = 6.37, upper whisker = 6.89, FASTS = Yes, min = 5.48, max = 7.00, lower whisker = 5.48, 25th% = 5.90, median = 6.32, 75th% = 6.66, upper whisker = 7.00. For *GPI* FASTS = No, min = 6.97, max = 10.51, lower whisker = 6.97, 25th% = 8.08, median = 8.19, 75th% = 9.08, upper whisker = 10.51, FASTS = Yes, min = 7.45, max = 8.91, lower whisker = 7.45, 25th% = 7.60, median = 7.87, 75th% = 8.45, upper whisker = 8.91. For *PCGF1* FASTS = No, min = 3.86, max = 5.93, lower whisker = 3.86, 25th% = 4.44, median = 4.55, 75th% = 4.95, upper

whisker = 5.32, FASTS = Yes, min = 4.38, max = 5.19, lower whisker = 4.38, 25th% = 4.38, median = 4.59, 75th% = 4.99, upper whisker = 5.19 (g) Spearman correlations of patient derived cell line age against FAS gene expression, coming from data in CCLE. *ACACA* is not included in (g) as it was not found in the CCLE expression data used in prior analysis. Shaded areas in (c),(d), (e), and (g) represent 95% CI of fitted line.

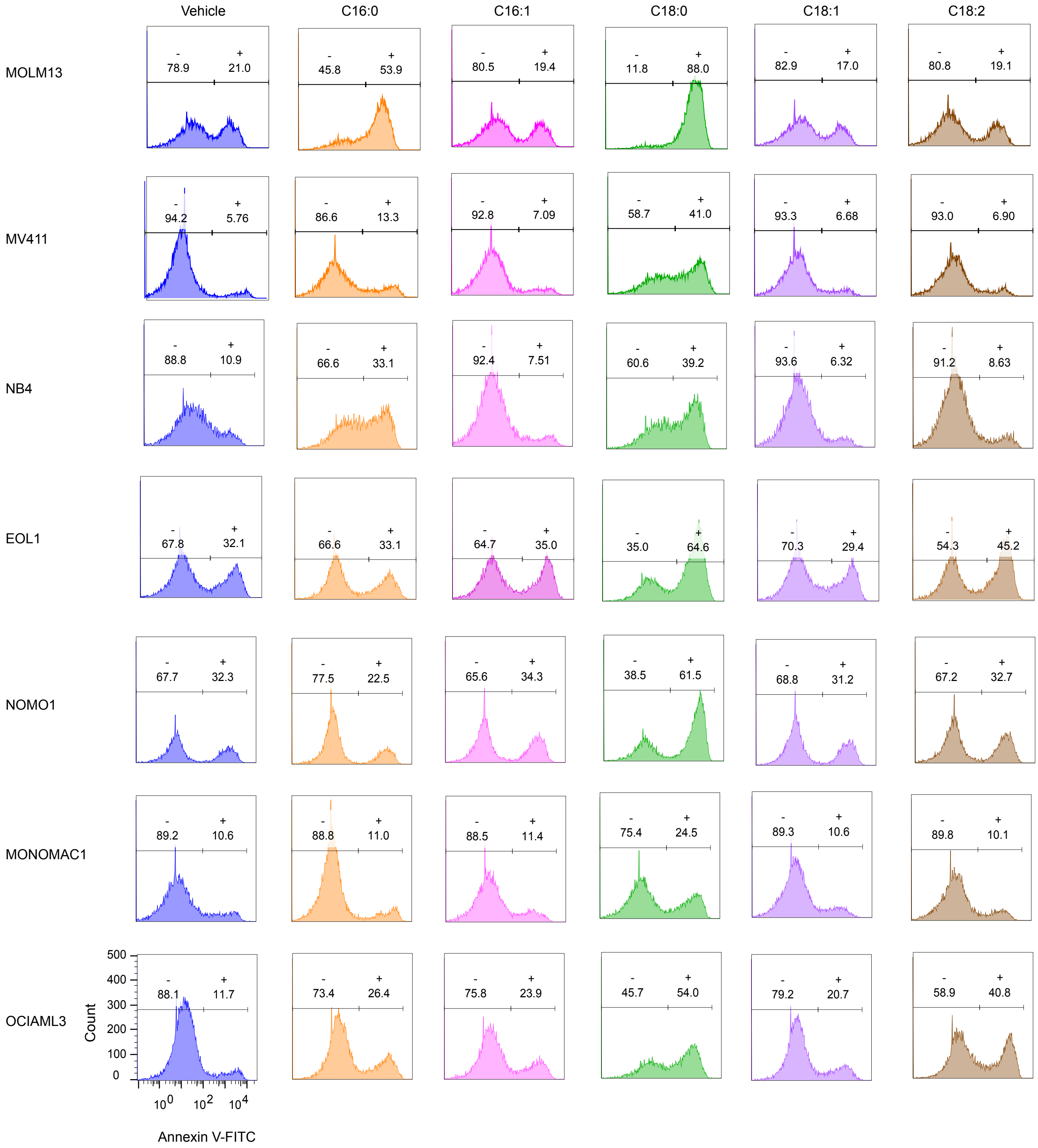

Vehicle
C16:0
C16:1
C18:0
C18:1
C18:2
MOLM13
- 78.9 + 21.0
- 45.8 + 53.9
- 80.5 + 19.4
- 11.8 + 88.0
- 82.9 + 17.0
- 80.8 + 19.1
MV411
- 94.2 + 5.76
- 86.6 + 13.3
- 92.8 + 7.09
- 58.7 + 41.0
- 93.3 + 6.68
- 93.0 + 6.90
NB4
- 88.8 + 10.9
- 66.6 + 33.1
- 92.4 + 7.51
- 60.6 + 39.2
- 93.6 + 6.32
- 91.2 + 8.63
EOL1
- 67.8 + 32.1
- 66.6 + 33.1
- 64.7 + 35.0
- 35.0 + 64.6
- 70.3 + 29.4
- 54.3 + 45.2
NOMO1
- 67.7 + 32.3
- 77.5 + 22.5
- 65.6 + 34.3
- 38.5 + 61.5
- 68.8 + 31.2
- 67.2 + 32.7
MONOMAC1
- 89.2 + 10.6
- 88.8 + 11.0
- 88.5 + 11.4
- 75.4 + 24.5
- 89.3 + 10.6
- 89.8 + 10.1
OCIAML3
- 88.1 + 11.7
- 73.4 + 26.4
- 75.8 + 23.9
- 45.7 + 54.0
- 79.2 + 20.7
- 58.9 + 40.8
Count
500
400
300
200
100
0
10^0
10^2
10^4
Annexin V-FITC

Figure S9. Sample flow cytometry plots. A representative flow cytometry data used to create bar graphs shown in figure 5b-c. Rows indicate which specific cell lines from figure 5b-c are used, and columns represent the various conditions used. Each plot represents a single dot from figure 5b-c.

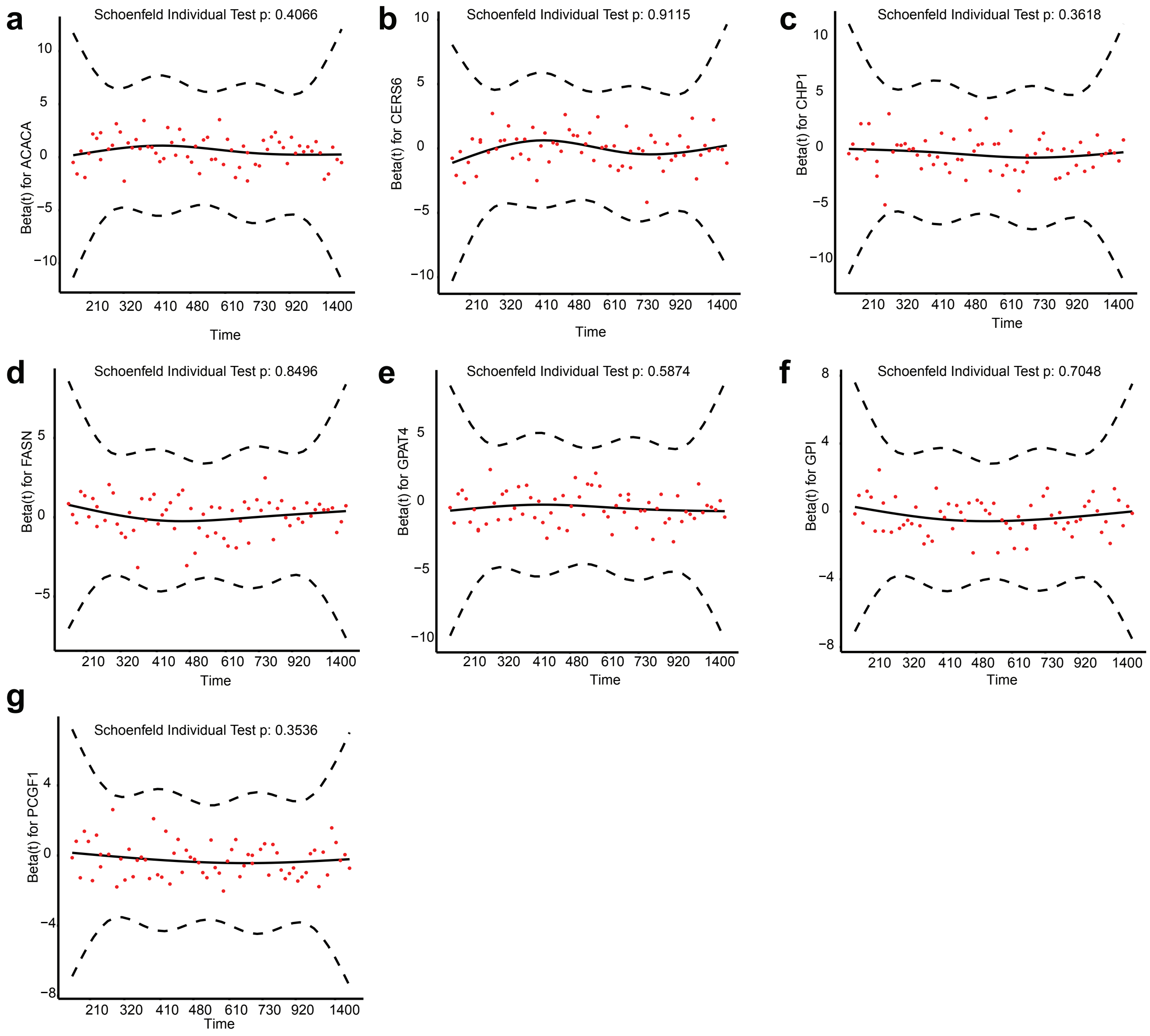
a
Schoenfeld Individual Test p: 0.4066
Beta(t) for ACACA
Time
b
Schoenfeld Individual Test p: 0.9115
Beta(t) for CERS6
Time
c
Schoenfeld Individual Test p: 0.3618
Beta(t) for CHP1
Time
d
Schoenfeld Individual Test p: 0.8496
Beta(t) for FASN
Time
e
Schoenfeld Individual Test p: 0.5874
Beta(t) for GPAT4
Time
f
Schoenfeld Individual Test p: 0.7048
Beta(t) for GPI
Time
g
Schoenfeld Individual Test p: 0.3536
Beta(t) for PCGF1
Time
210 320 410 480 610 730 920 1400

Figure S10. Testing the Cox Proportional Hazards Assumption. Assessing the Cox proportional hazards assumption with Schoenfeld tests of all genes in Figure 6d; (a) *ACACA*, (b) *CERS6*, (c) *CHP1*, (d) *FASN*, (e) *GPAT4*, (f) *GPI*, (g) *PCGF1*.

Supplemental References

1. Aregger, M. *et al.* Systematic mapping of genetic interactions for de novo fatty acid synthesis identifies C12orf49 as a regulator of lipid metabolism. *Nature Metabolism* **2**, 499–513 (2020).
