# Supplementary material for: Discovery of putative tumor suppressors from CRISPR screens reveals rewired lipid metabolism in acute myeloid leukemia cells
Source: Nat Commun. 2021 Nov 11;12:6506. doi: 10.1038/s41467-021-26867-8 (PMC8586352; doi:10.1038/s41467-021-26867-8)
Supplement: Supplementary file 10 — Reporting Summary [file 41467_2021_26867_MOESM10_ESM.pdf]

nature portfolio

Corresponding author(s): Traver Hart

Last updated by author(s): Oct 26, 2021

Reporting Summary

Nature Portfolio wishes to improve the reproducibility of the work that we publish. This form provides structure for consistency and transparency in reporting. For further information on Nature Portfolio policies, see our Editorial Policies and the Editorial Policy Checklist.

Statistics

For all statistical analyses, confirm that the following items are present in the figure legend, table legend, main text, or Methods section.

n/a	Confirmed	
☐	☒	The exact sample size (*n*) for each experimental group/condition, given as a discrete number and unit of measurement
☒	☐	A statement on whether measurements were taken from distinct samples or whether the same sample was measured repeatedly
☐	☒	The statistical test(s) used AND whether they are one- or two-sided<br>*Only common tests should be described solely by name; describe more complex techniques in the Methods section.*
☐	☒	A description of all covariates tested
☐	☒	A description of any assumptions or corrections, such as tests of normality and adjustment for multiple comparisons
☐	☒	A full description of the statistical parameters including central tendency (e.g. means) or other basic estimates (e.g. regression coefficient) AND variation (e.g. standard deviation) or associated estimates of uncertainty (e.g. confidence intervals)
☐	☒	For null hypothesis testing, the test statistic (e.g. *F*, *t*, *r*) with confidence intervals, effect sizes, degrees of freedom and *P* value noted<br>*Give P values as exact values whenever suitable.*
☒	☐	For Bayesian analysis, information on the choice of priors and Markov chain Monte Carlo settings
☒	☐	For hierarchical and complex designs, identification of the appropriate level for tests and full reporting of outcomes
☐	☒	Estimates of effect sizes (e.g. Cohen's *d*, Pearson's *r*), indicating how they were calculated

Our web collection on statistics for biologists contains articles on many of the points above.

Software and code

Policy information about availability of computer code

Data collection

Code Availability
Genetic Interaction (enCas12a) code notebooks pertaining to figure 4, and supplemental figure 6 can be found at https://github.com/PeterDeWeirdt/FASTS. Code pertaining to all figures except for Figure 4, Supplemental Figure 6, and 9 is available at: https://doi.org/10.6084/m9.figshare.16786063 Additional analysis code (primarily co-occurrence network, mixed z-score metrics, dPCC correlation, and clinical analysis) is available at: https://doi.org/10.6084/m9.figshare.16786078.v1

Data Availability
Genetic Interaction (enCas12a) data pertaining to figure 4, and supplemental figure 6 can be found at https://github.com/PeterDeWeirdt/FASTS. Figure 5 b-c data can be found within the source data file. Cytoscape network files of PSG network (Figure 2 and Supplemental Figure 4) can be found at: https://doi.org/10.6084/m9.figshare.16746052.v1. Relevant data for figures, including gene Mix Z-score evaluation, fisher edge calculations, dPCC scoring metrics, and other screen metric comparisons, can be found at: https://doi.org/10.6084/m9.figshare.16746040.v1.

External data used in this study includes the screening set coming from the Avana 2020q4 release, and CCLE genetic expression, mutation, and copy number data that can be found at www.depmap.org;screening data used from Project Score that can be found at https://depmap.sanger.ac.uk/; Cell Model Passports data was used in screening data comparison and can be found at https://cellmodelpassports.sanger.ac.uk/; the cancer gene census used to define oncogenes and tumor suppressors that can be found at https://cancer.sanger.ac.uk/census; absolute gene copy number values from cell lines obtained the cBioPortal database at https://www.cbioportal.org/; HumanNet data used for network comparisons can be found at https://www.inetbio.org/humannet/; the Xena database was used in acquiring specific data related to the TCGA LAML, TARGET AML, and BeatAML datasets and can be found at https://xenabrowser.net/; and additional BeatAML analysis was taken directly from Tyner et al. Nature 2018 publication.

The results published here are in part based upon data generated by the Therapeutically Applicable Research to Generate Effective Treatments (TARGET) initiative, phs000218, managed by the NCI. The data used for this analysis are available at dbGaP Study Accession:

phs000465.v19.p8 [https://www.ncbi.nlm.nih.gov/projects/gap/cgi-bin/study.cgi?study_id=phs000465.v19.p8]. Information about TARGET can be found at http://ocg.cancer.gov/programs/target.

Data analysis

Mixed Z-scoring, analysis using scoring metric, co-occurrence network, and survival analysis was conducted in R version 4.0.4. dPCC correlation analysis, including empirical calculations were conducted in Python 3.8.2, using the packages SciPy, NumPy, Matplotlib, and pandas.

R packages tidyverse, data.table, and knitr were used for figure generation, data manipulation, and general R functions; mixtools, permute, and PRROC were used for data simulations present in figures and evaluation; biomaRt, and org.Hs.eg.db were used in integrating data types; cowplot, ggbeeswarm, annotate, RColorBrewer, ComplexHeatmap, gplots, ggpubr, grid, circlize, ggthemes, ggExtra, patchwork, and ggplot2, were used for figure aesthetics and generation. R packages survival and survminer were used for survival analysis and figure generation. Analysis related to Kaplan Meier and patient stratification was done in python version 3.8.5 using the packages pandas, numpy, and scipy for statistical functions and data manipulation, seaborn, plotly, and matplotlib for figure aesthetics and generation, and lifelines for both statistical analysis and figure generation.

Analysis of enCas12a multiplex genetic screens was conducted in R 4.0.0 and Python 3.8.3. Code for this analysis is available at https://github.com/PeterDeWeirdt/FASTS. R packages tidyverse and tidygraph were used for data manipulation and ggraph was used for graph visualization. Python packages SciPy, NumPy, Matplotlib, pandas, statsmodels, plotnine were used for analysis and visualization. The Custom package gnt was used to calculate genetic interaction scores and gpplot was used to generate point density plots.

For manuscripts utilizing custom algorithms or software that are central to the research but not yet described in published literature, software must be made available to editors and reviewers. We strongly encourage code deposition in a community repository (e.g. GitHub). See the Nature Portfolio guidelines for submitting code & software for further information.

Data

Policy information about availability of data

All manuscripts must include a data availability statement. This statement should provide the following information, where applicable:

- Accession codes, unique identifiers, or web links for publicly available datasets
- A description of any restrictions on data availability
- For clinical datasets or third party data, please ensure that the statement adheres to our policy

Genetic Interaction (enCas12a) data pertaining to figure 4, and supplemental figure 6 can be found at https://github.com/PeterDeWeirdt/FASTS. Figure 5 b-c data can be found within the source data file. Cytoscape network files of PSG network (Figure 2 and Supplemental Figure 4) can be found at: https://doi.org/10.6084/m9.figshare.16746052.v1. All other relevant data for figures, can be found at: https://doi.org/10.6084/m9.figshare.16746040.v1.

Field-specific reporting

Please select the one below that is the best fit for your research. If you are not sure, read the appropriate sections before making your selection.

☒ Life sciences ☐ Behavioural & social sciences ☐ Ecological, evolutionary & environmental sciences

For a reference copy of the document with all sections, see nature.com/documents/nr-reporting-summary-flat.pdf

Life sciences study design

All studies must disclose on these points even when the disclosure is negative.

Sample size

No sample sizes were determined. Sample sizes are based on available data.

Data exclusions

DPCC Analysis - Cell line screens that performed poorly (Cohen's D < 2.5 or recall of known core essential genes < 60%) were excluded, leaving 659 cell lines.
Clinical Analysis - We filtered out samples from recurrent patients, and chose to analyze patient expression profiles from primary tumor samples.

Replication

We attempted to replicate specific proliferation suppressor hit calls from the Broad DepMap screen set against the Sanger DepMap screen set. We observed evidence that some hit calls were consistent in the Sanger DepMap set, however due to experimental conditions, discussed in the manuscript, we were unable to fully verify all of our hit calls. No other viable screen set could be used for replication of analysis.

Randomization

No human or animal subjects were used in the experimental conditions.

Blinding

No human or animal subjects were used in the experimental conditions. Published patient data was reanalyzed.

Reporting for specific materials, systems and methods

We require information from authors about some types of materials, experimental systems and methods used in many studies. Here, indicate whether each material, system or method listed is relevant to your study. If you are not sure if a list item applies to your research, read the appropriate section before selecting a response.

Materials & experimental systems

n/a	Involved in the study
☒	☐ Antibodies
☐	☒ Eukaryotic cell lines
☒	☐ Palaeontology and archaeology
☒	☐ Animals and other organisms
☒	☐ Human research participants
☒	☐ Clinical data
☒	☐ Dual use research of concern

Methods

n/a	Involved in the study
☒	☐ ChIP-seq
☐	☒ Flow cytometry
☒	☐ MRI-based neuroimaging

Eukaryotic cell lines

Policy information about cell lines

Cell line source(s)

MDACC:Cell lines used include: MOLM13 (Fisher #NC0442994), NOMO1 (Fisher #NC1515509), MV411 (ATCC #CRL-9591), MONOMAC1 (DSMZ #ACC-252), OCIAML3 (DSMZ #ACC-582), NB4 (DSMZ #ACC-207), and EOL1 (DSMZ #ACC-386).

BROAD:MOLM13 and NOMO1 cells screened with the Cas12a-mediated genetic interaction library at the Broad Institute were obtained from the Cancer Cell Line Encyclopedia.

Authentication

MDACC: Cell lines were authenticated using STR profiling at the M.D. Anderson Cancer Center Characterized Cell Line Core prior to experimentation.

Mycoplasma contamination

BROAD: All cell lines were routinely tested for mycoplasma contamination and were maintained without antibiotics except during screens, when the media was supplemented with 1% penicillin/streptomycin.

MDACC:To ensure absence of mycoplasma contamination, PlasmoTest Mycoplasma Detection Kit (Invivogen #rep-pt1) was used monthly. Presence of contaminants was indicated by a colorimetric test. Cell lines were verified mycoplasma free by the absence of color change after overnight incubations in conjunction with positive and negative controls.

Commonly misidentified lines
(See ICLAC register)

No commonly misidentified cells were used in this study.

Flow Cytometry

Plots

Confirm that:

☒ The axis labels state the marker and fluorochrome used (e.g. CD4-FITC).

☒ The axis scales are clearly visible. Include numbers along axes only for bottom left plot of group (a 'group' is an analysis of identical markers).

☒ All plots are contour plots with outliers or pseudocolor plots.

☒ A numerical value for number of cells or percentage (with statistics) is provided.

Methodology

Sample preparation

Cells were treated for 48 h with either a vehicle control (0.1% BSA + 0.1 % EtOH), or the saturated fatty acids C16:0 and C18:0, MUFA C16:1 and 18:1, or PUFA C18:2 at 200 mM. Flow cytometry was used to measure cell death using FITC annexin V Detection Kit I (556547, BD Biosciences) and propidium iodide (PI) (P3566, Life Technologies) according to the manufacturer's instructions. In brief, after 48 h. of fatty acid treatment cells were transferred to a deep well plate, centrifuged at 500 xg for five minutes, the media was removed by flipping the plate over. Cells were then washed with 1 ml of 1X PBS centrifuge for five min at 500 xg. After removing 1X PBS cells were resuspended in 1X binding buffer and stained simultaneously with FITC-labeled annexin V and propidium iodide (PI) for 15 minutes at room temperature. For every cell type, to set up flow cytometry parameters of compensation and quadrants, we used unstained cells, cells stained with FITC Annexin V (No PI) and cells stained with PI (No FITC Annexin V). All measurements were done using the same parameters to be able to compare among cell lines.

Instrument

FACSCelesta Cell Analyzer
Software Version: BD FACSDiva 8.0.1.1
Laser: 488
Filter 530/30 BP: Alexa Fluor 488, FITC,
Laser: 561
Filter 610/20 BP: PI

Sample Acquisition: 12mm x 75mm polystyrene tubes (Controls), 96, well plates (treated samples)

Software	Flow cytometry data collected with FACSCelesta Cell Analyzer, software version: BD FACSDiva 8.0.1.1. Flow cytometry data were analyzed using FlowJo 10.5.3.
Cell population abundance	The total stopping gate was set up at 25,000 cells. From total cells, the next gate was single cells. From single cells, cells were counted in relationship to the percent of parent population. Population was divided in four quadrants and reported as a percentage. Percentage of live cells (negative for PI and negative for annexin V), early apoptosis (Annexin V+ and propidium iodide -), late apoptosis (PI + and Annexin V +), and necrosis (PI+ and Annexin V-)
Gating strategy	All cells: On the X-axis FSC-A and on the Y-axis SSC-A both from 0 to 250,000<br>Singlets: On the X-axis FSC-H and on the Y-axis FSC-A both from 0 to 250,000<br><br>Two parameter density plots for further analysis: X-axis Alexa Fluor 388-A/FITC (Annexin V stain) and Y-axis PI-A (Propidium iodide stain) both from 0 to 10E5<br><br>Q1: PI+, Annexin V-<br>Q2: PI+, Annexin +<br>Q3: PI-, Annexin -<br>Q4: PI-, Annexin +<br><br>Single parameter histograms for identifying cells stained with either PI or Annexin V were used.<br>Annexin V histogram: X-axis Alexa Fluor 488-A/FITC (0 to 1E5 log scale), Y-axis Count<br>Negative control: Unstain for PI or Annexin V X-axis histogram from 0 to 10E2.5 to 10E3<br>Annexin+ or PI+: X-axis histogram from 10E3 to 10E5, Y axis Count

☒ Tick this box to confirm that a figure exemplifying the gating strategy is provided in the Supplementary Information.
